# First Characterization and Description of *Aspergillus* Series *Versicolores* in French Bioaerosols

**DOI:** 10.3390/jof7080676

**Published:** 2021-08-21

**Authors:** Antoine Géry, Jean-Philippe Rioult, Natacha Heutte, Virginie Séguin, Julie Bonhomme, David Garon

**Affiliations:** 1 ToxEMAC-ABTE, Centre F. Baclesse, Unicaen and Unirouen, Normandie University, 14000 Caen, France; antoine.gery@unicaen.fr (A.G.); jean-philippe.rioult@unicaen.fr (J.-P.R.); virginie.seguin@unicaen.fr (V.S.); bonhomme-j@chu-caen.fr (J.B.); 2 CETAPS, UFR Sciences et Techniques des Activités Physiques et Sportives Unirouen, Normandie University, 76000 Rouen, France; natacha.heutte@univ-rouen.fr; 3 Service de Microbiologie, Centre Hospitalier Universitaire de Caen, 14000 Caen, France

**Keywords:** *mold*, *Versicolores* series, bioaerosols

## Abstract

Air quality can be altered by fungal contaminants suspended in the air, forming bioaerosols. *Aspergilli* section *Nidulantes* series *Versicolores* are recurrent in bioaerosols and are mainly responsible for allergies and asthma aggravation. Phylogenetic studies recently identified 12 new species within this series. This study is the first to identify species of *Aspergillus* series *Versicolores* in French bioaerosols and to characterize them macroscopically, microscopically and molecularly. Bioaerosols were collected in a cancer treatment center, in contaminated homes and in agricultural environments. A total of 93 isolates were cultured on selective media, observed by optical microscopy and identified by *benA* amplification before sequencing. The field data (temperature and relative humidity) were statistically tested to explore the ecology of these species. Eight species were identified from bioaerosols: *Aspergillus creber* and *A. jensenii*, which represent more than 80% of the isolates, and *A. protuberus*, *A. puulaauensis*, *A. sydowii*, *A. tabacinus*, *A. amoenus* and *A. fructus*. *Aspergilli* series *Versicolores* are distributed differently depending on the sampling site and climatic determinants. *Aspergillus protuberus* was found in bioaerosols collected under significantly lower relative humidity (*p* = 3.899 × 10^−4^). Characterization and repartition of these isolates belonging to the *Versicolores* series constitute an important step to better assess exposure to fungal bioaerosols.

## 1. Introduction

Over the last decades, air quality has become a major public health issue, especially in industrialized countries. It is estimated that the population of these countries (including France) spends up to 90% of its time indoors [1,2], which has been exacerbated by the successive lockdowns related to the SARS-COV2 health crisis. According to the WHO [3], 30–50% of buildings in Europe have moisture problems that facilitate mold growth and bioaerosols’ formation, leading to impaired indoor air quality [4]. These molds are visible in 14% to 20% of French housing [5]. This degradation of indoor air quality in homes has an impact on the quality of life and can lead to economic consequences estimated at 19 billion euros per year for France in terms of health expenses and renovation costs [6]. Some of these molds are known to aggravate asthma [7], cause allergies [8] and sometimes induce infections in immunocompromised individuals [9,10]. Some micromycetes are also capable of producing mycotoxins that may possess cytotoxic [11,12] and/or genotoxic properties [13,14].

Molds of the genus *Aspergillus* belonging to the series *Versicolores* section *Nidulantes* are found in hypersaline water [15], in soil [16], in various foods and feeds [17] and in indoor environments, where they are estimated to be isolated from more than 70% of bioaerosols [18]. These species are especially known to cause allergies [19], aggravate asthma [20] and be involved in the sick building syndrome [21]. They are also found more sporadically as agents responsible for onychomycosis [22] or pulmonary aspergillosis [23]. More recently, their presence has been highlighted as an explanatory variable for various symptoms experienced by the inhabitants of mold-damaged homes, such as dizziness, fever, headache, itching or expectorations [18]. These are also known to produce sterigmatocystin [24], a mycotoxin involved in the aflatoxin biosynthetic pathway and which is recognized as a potential carcinogen by the IARC (group 2B) [25]. The species *Aspergillus versicolor* was considered until a few years ago as the most recurrent species of the *Versicolores* series present in bioaerosols. Recent phylogenetic studies [26,27] have led to taxonomic revisions that have revealed a total of 12 new species within the *Versicolores* series. Among these new species, *Aspergillus creber* is considered as the most frequent species and was misidentified as *Aspergillus versicolor* [28]. The few studies on the relative abundance of species of the *Versicolores* series in different matrices seem to confirm this hypothesis [29,30]. We also know that the different species in the *Versicolores* series do not all produce the same metabolites and are not as frequent in human pathology as each other [31].

In this study, we characterize and describe 93 environmental isolates of *Aspergillus* series *Versicolores* collected from French bioaerosols in order to evaluate their diversity and contribute to fungal exposure to *Aspergillus* series *Versicolores*.

## 2. Material and Methods

### 2.1. Sampling

Bioaerosols were collected indoors from a cancer treatment center (Centre François Baclesse, Caen, France) (*n* = 24) [32] and in contaminated homes (*n* = 65) [18,33], and outdoor in agricultural environments (silage and hay) (*n* = 4) [34,35]. During collection, a Grimm particle counter (Model 1.108, Grimm Technologies, Inc., Douglasville, GA, USA) was used to measure temperature and relative humidity every 6 s. The sampling of bioaerosols in contaminated homes was carried out during two different campaigns: the first one in *Serpula lacrymans*-damaged homes [33] and the second in mold-damaged homes [18].

Samples were cultured on Malt Extract Agar medium with 0.02% chloramphenicol (Cooper, Melun, France) (MEA+). Plates were incubated at 25 °C and checked daily. Each fungal colony was isolated and purified on the same medium. All *Aspergillus* isolates belonging to the *Versicolores* series (*n* = 93) were stored on slant agar at −4 °C and in a cryoprotective agent composed of sterile water (Fresenius Kabi AG, Bad Homburg, Germany) and 10% glycerol (Carlo Erba, Val-de-Reuil, France) at −80 °C before characterization.

### 2.2. Molecular Characterization

DNA extraction was performed using a modified protocol of the Nucleospin™ Plant II kit (Macherey-Nagel, Duren, Germany). Fungal colony was introduced in a 2 mL microtube with glass beads. The microtube underwent two incubation cycles of 15 min at 80 °C and then 15 min at −80 °C. It was placed into a Qiagen Tissue Lyser with 400 μL of lysis buffer PL1 (Macherey-Nagel, Duren, Germany) for 15 min at 20 Hz, and incubated with 10 µL of RNAse (Macherey-Nagel, Duren, Germany) and 20 μL of proteinase K at 10 mg/mL (Sigma-Aldrich, St. Louis, MO, USA) at 65 °C for 15 min. Then, 400 μL of chloroform (Sigma-Aldrich, St. Louis, MO, USA) was added to the mixture. The aqueous phase was recovered and extracted using the precipitation kit and washing buffer according to the protocol described by the supplier.

DNA was purified using the NucleoSpin gDNA Clean-up kit (Macherey-Nagel, Duren, Germany) following the instructions of the manufacturer. For each isolate, quantification and quality of purified DNA were realized using a NanoDrop 2000 spectrophotometer (Thermo Fisher Scientific, Waltham, MA, USA) [36].

Molecular characterization was performed by amplification of the beta-tubulin gene (*benA*) using Bt2a/Bt2b (5′-GGTAACCAAATCGGTGCTGCTTTC-3′/5′-ACCCTCAGTGTAGTGACCCTTGGC-3′) primers [27,28]. The end-point PCR program included: a hold stage at 94 °C for 5 min, a PCR stage (94 °C for 30 s; 55 °C for 45 s; 72 °C during 90 s) repeated for 35 cycles and another hold stage at 72 °C for 5 min. PCR products were then sequenced by GATC (Eurofins, Hamburg, Germany). Sequences obtained were compared using BLAST (Basic Local Alignment Search Tool, NCBI) to the reference sequences of the 17 *Aspergillus* species of the *Versicolores* series (accession numbers: JN853946, JN853963, JN853980, EF652264, EF652273, KJ775086, LC000552, JN854007, KU613371, EF652284, JN853979, JN853970, EF652274, EF652302, JN853976, JN854003, EF652266) [27] and to the sequences of the CoreNucleotide, dbEST and dbGSS databases. Identification was considered reliable only by having a query cover ≥ 98% and a % ID ≥ 99%.

### 2.3. Macroscopic Characterization

Macroscopic characterization was performed by culture of the *Versicolores* isolates on four different media: MEA without chloramphenicol (MEA), Czapek medium (CZ), Czapek Yeast Autolysate Agar (CYA25) and Czapek Yeast Extract Agar with 20% sucrose (CY20S) at 25 °C and on CYA at 37 °C (CYA37). Conidia from 7- to 14-day-old cultures were mixed in soft agar (0.2% agar + 0.05% Tween 80 (Sigma-Aldrich, St. Louis, MO, USA)). Each medium was inoculated in three points equidistant from the center with 2 µL of this spore suspension. After 7 days of culture, the diameters, colors (given as hex color codes), textures and reliefs of the colonies were noted. After 14 days of culture, the presence or absence of exudate and diffusible pigment was noted [37,38].

### 2.4. Microscopic Characterization

Microscopic characterization was performed with an optical microscope Olympus CX31 (Olympus, Tokyo, Japan) after 10 days of culture on MEA, or on CY20S in case of low sporulation on MEA. For each isolate, the appearance and size of vesicles, the size of metulae, phialides and conidia, the texture of conidia, the color of fungal structures and the presence or absence of Hülle cells were specified [37,38].

### 2.5. Statistical Analysis

Descriptive statistics were calculated to provide information on isolates, and environmental data were subjected to the Mann–Whitney test. Only results at *p* < 0.05 were considered statistically significant. All statistical analyses were realized using XLSTAT (Addinsoft, Paris, France).

## 3. Results

### 3.1. Relative Abundance in French Bioaerosols

Among all the mold species found in the different bioaerosols, *Aspergilli* series *Versicolores* were ranked fifth in bioaerosols from the cancer treatment center, first in *Serpula lacrymans*- and mold-damaged homes and third in agricultural environments, with concentrations ranging from 2.48 CFU/m^3^ to 3.44 × 10^5^ CFU/m^3^ (mean: 9.30 × 10^3^ CFU/m^3^). *Aspergilli* series *Versicolores* were associated with 109, 100 and 28 other fungal species in bioaerosols collected from the cancer treatment center, *Serpula* lacrymans- and mold-damaged homes, and in agricultural environments, respectively. The most represented genera in all these environments were *Aspergillus (A. fumigatus*, *A. niger*, *A. melleus*, *A. flavus*, *A. pseudoglaucus*), *Penicillium* (*P. chrysogenum*, *P. brevicompactum*, *P. crustosum*) and *Cladosporium* (*C. cladosporioides*, *C. herbarum*, *C. sphaerospermum*) [18,32,33,34].

**Figure 1:**
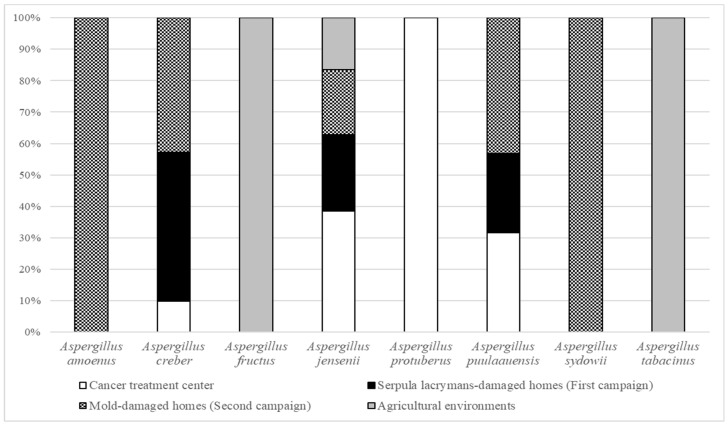
Relative abundances of *Aspergillus* species in the *Versicolores* series depending on bioaerosols’ sampling location.

As shown in Figure 1, among all the isolates collected from French bioaerosols (*n* = 93), we were able to molecularly identify 8 species of the *Versicolores* series: *Aspergillus creber* (43%) ranked first, followed by *A. jensenii* (40%), *A. protuberus* (7%), *A. puulaauensis* (4%), *A. sydowii* (2%), *A. tabacinus* (2%), *A. amoenus* (1%) and *A. fructus* (1%). Bioaerosols collected in the cancer treatment center (*n* = 24) contained more *A. jensenii* (58%), followed by *A. protuberus* (25%), *A. creber* (13%) and *A. puulaauensis* (4%). *A. protuberus* was only found in bioaerosols collected in the cancer treatment center. Bioaerosols collected in *Serpula lacrymans*- and mold-damaged homes (*n* = 65) were mainly composed of *A. creber* (57%) and *A. jensenii* (34%). *A. puulaauensis* (5%), *A. sydowii* (3%) and *A. amoenus* (2%) were also present. Among the four isolates obtained from bioaerosols collected in agricultural environments, we identified two isolates of *A. tabacinus*, one isolate of *A. jensenii* and one isolate of *A. fructus*. *Aspergillus tabacinus* and *A. fructus* were only found in bioaerosols collected in agricultural environments. *A. jensenii* was the only species found in all environments. We also observed a similar distribution of *Versicolores* species within the contaminated homes independently in the sampling campaign with the highest species richness.

### 3.2. Environmental Data

The temperature measured in these different environments varied between 9.4 and 26.1 °C, and the relative humidity between 23.8% and 74.3% (Table 1). *Aspergillus amoenus*, *A. fructus* and *A. tabacinus* were found in bioaerosols collected at low temperatures, while *A. protuberus* and *A. sydowii* were found in bioaerosols collected at higher temperatures. *Aspergillus fructus*, *A. protuberus* and *A. tabacinus* were also found in bioaerosols collected with low relative humidity, while *A. amoenus*, *A. creber* and *A. sydowii* were found in bioaerosols collected with high relative humidity. It should be noted that only *Aspergillus protuberus* was found in bioaerosols collected in the cancer treatment center with a significantly lower relative humidity than in bioaerosols not containing *Aspergillus protuberus* (*p* = 3.899 × 10^−4^).

### 3.3. Description of the Versicolores Series Species

The culture on four selective media and the microscopic observations of ninety-three fungal isolates provide full details on these eight *Aspergillus* species in the series *Versicolores* (Table 2).

#### 3.3.1. *Aspergillus amoenus*

**Figure 2:**
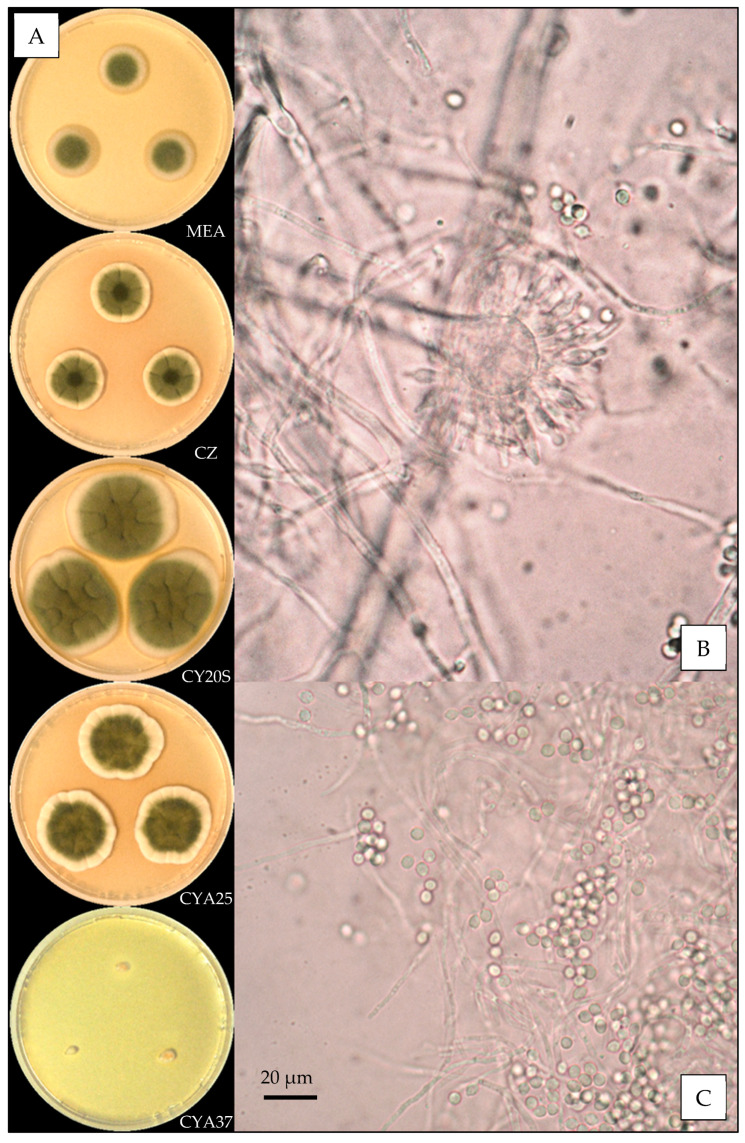
*Aspergillus amoenus*: (**A**) from top to bottom, colonies on MEA, CZ, CY20S, CYA25 and CYA37, 14 days. (**B**) Conidial head and (**C**) conidia (1000×).

Description based on *n* = 1 isolate (Figure 2).

**Macroscopic characteristics**

Colony diameters at 7 days, in mm, were: MEA 11–15, CZ 12–15, CY20S 23–27, CYA25 20–23 and CYA37 0–9. 

On MEA, there were circular and flat colonies with an entire margin, conidia greyish green (from #8e9066 to #6d714d), mycelium was white to buff, reverse uncolored to yellow brown (#c1a457), texture was velutinous to granular and there was no exudate or soluble pigment. On CZ, there were circular and flat colonies flat with an entire margin, conidia were pale greyish green to greyish green (from #d9cd97 to #a8a975), mycelium were white to buff, reverse yellow brown (from #c89847 to #c59951), and texture was velutinous to granular; when present, exudate was uncolored or brownish orange to brownish red (from #63512a to #4b210f), and when present, soluble pigment was orange (#ba8d2e). On CY20S, circular and umbonate colonies radially sulcate with an entire margin, conidia grey to greyish green (from #a8a687 to #9ea067), mycelium was white to buff, reverse brownish yellow to orange (from #d1b236 to #c29833), texture was velutinous and there was no exudate or soluble pigment. On CYA25, circular to irregular and flat colonies radially sulcate with an entire margin, conidia were dull green to greyish green (from #a6b27a to #989467), mycelium was white to buff, reverse brownish yellow to orange (from #d1b236 to #c29833), texture was velutinous to granular and exudate was uncolored to brownish orange (#be985d); when present, soluble pigment was brownish orange (#c59145). On CYA37, there were dense, convex and crateriform colonies with an entire margin, no conidiation with mycelium white to pale brown (#cdb489), reverse brown (#a27931), texture was velutinous and there was no exudate or soluble pigment.

**Microscopic characteristics**

Conidial heads radiate uncolored to greyish green vesicles expanding into pyriform vesicles 12–15 µm in diameter, biseriate, metulae of 4–6 µm covering all the vesicles and phialides of 5–6 µm. No diminutive conidial head was observed for *Aspergillus amoenus* isolates. Conidia were 3–4 µm in diameter, mostly globose to sub-globose, with finely or distinctly roughened walls. No globose Hülle cells or chlamydospores were observed.

#### 3.3.2. *Aspergillus creber*

**Figure 3:**
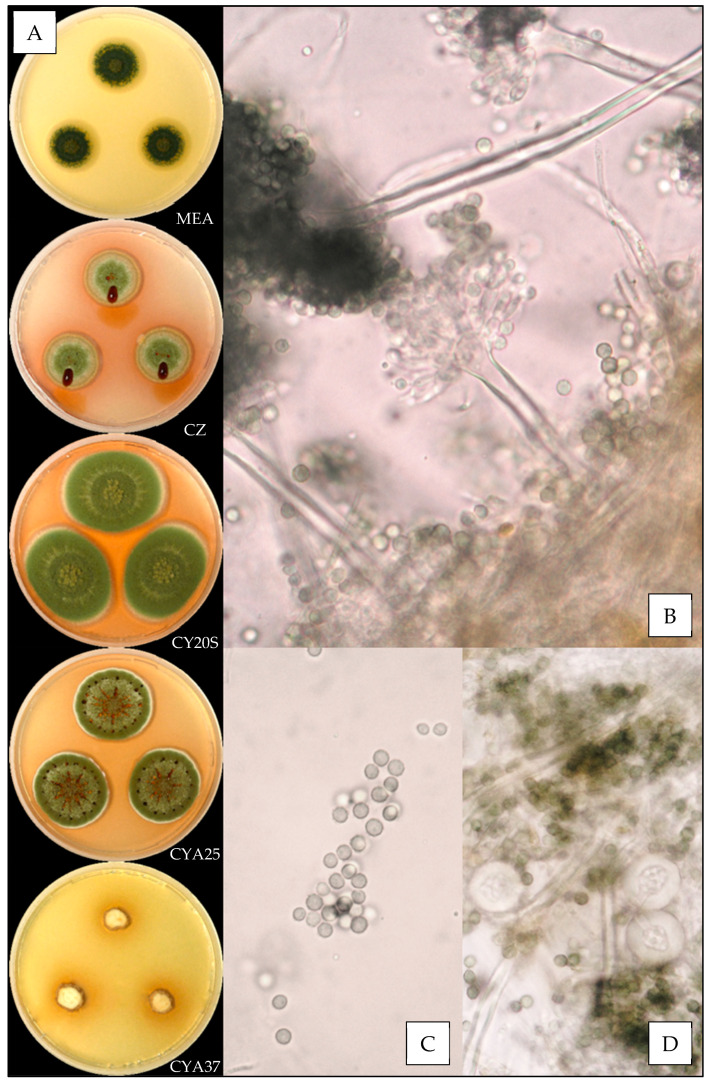
*Aspergillus creber*: (**A**) from top to bottom, colonies on MEA, CZ, CY20S, CYA25 and CYA37, 14 days. (**B**) Conidial head, (**C**) conidia and (**D**) globose Hülle cells (1000×).

Description based on *n* = 40 isolates (Figure 3).

**Macroscopic characteristics**

Colony diameters at 7 days, in mm, were: MEA 8–16 (–17), CZ (8–) 10–16 (–17), CY20S (18–) 19–26 (–28), CYA25 15–22 (–25) and CYA37 0–6 (–8).

On MEA, there circular and flat colonies with an entire margin, conidia yellow to dark green (from #e3bc25 to #52582a), mycelium was white to buff, reverse uncolored to orange (#eea620), texture was velutinous to granular and there was no exudate or soluble pigment. On CZ, there were circular and flat colonies with an entire margin, conidia were pale yellow or dull green to greyish green (#ffdc61 or #beb790 to #77723c), mycelium was white to buff, reverse orange to brownish red (from #be7329 to #602909), texture was velutinous to granular; when present, exudate was yellow, brownish red, brown or black (#ddbc72, #5e1701, # 804a0c or #100c03) and soluble pigment was orange pink to dark pink (from #dfa06b to #b63346). On CY20S, circular and flat colonies sometimes radially sulcate with an entire margin, conidia were beige to greyish green (from #e0bb74 to #6b6023), mycelium was white or buff to brownish orange (#905a21), reverse brownish orange to brownish red (from #cc9700 to #501a00), texture was velutinous; when present, rare exudate was yellow to brownish orange (#eccc53 to #894d20) and soluble pigment was dark red (#a53000). On CYA25, circular and flat colonies often radially sulcate with an entire margin, conidia were yellow beige or dull green to greyish green (#f2cb67 or #646945 to #76774e), mycelium was white to buff, reverse brownish orange (#d57a18), texture was velutinous to granular; when present, exudate was brownish with shades from yellow to red or black (#a3884a to #501c01 or #170d03), and when present, soluble pigment was dull orange to dark pink (#e8a052 to #8e1c24). On CYA37, when present, colonies were circular and flat with an entire margin, white to dull orange (#db9441), reverse brownish yellow to brown (#ca9329 to #6c4b0a), texture was velutinous and there was no exudate; when present, soluble pigment was brownish orange (#a46016).

**Microscopic characteristics**

Conidial heads radiate uncolored to greyish green vesicles expanding into pyriform vesicles of (3–) 5–13 (–15) µm in diameter, biseriate, metulae of (3–) 4–6 (–7) µm covering two thirds to all the vesicles and phialides of 4–6 (–8) µm. Diminutive conidial heads were observed for *Aspergillus creber* isolates. Conidia were of 2.5–4.5 µm in diameter, mostly globose to sub-globose, with finely or most of the time distinctly roughened walls. Hülle cells (12–14 µm) were found, but no chlamydospores were observed.

#### 3.3.3. *Aspergillus fructus*

**Figure 4:**
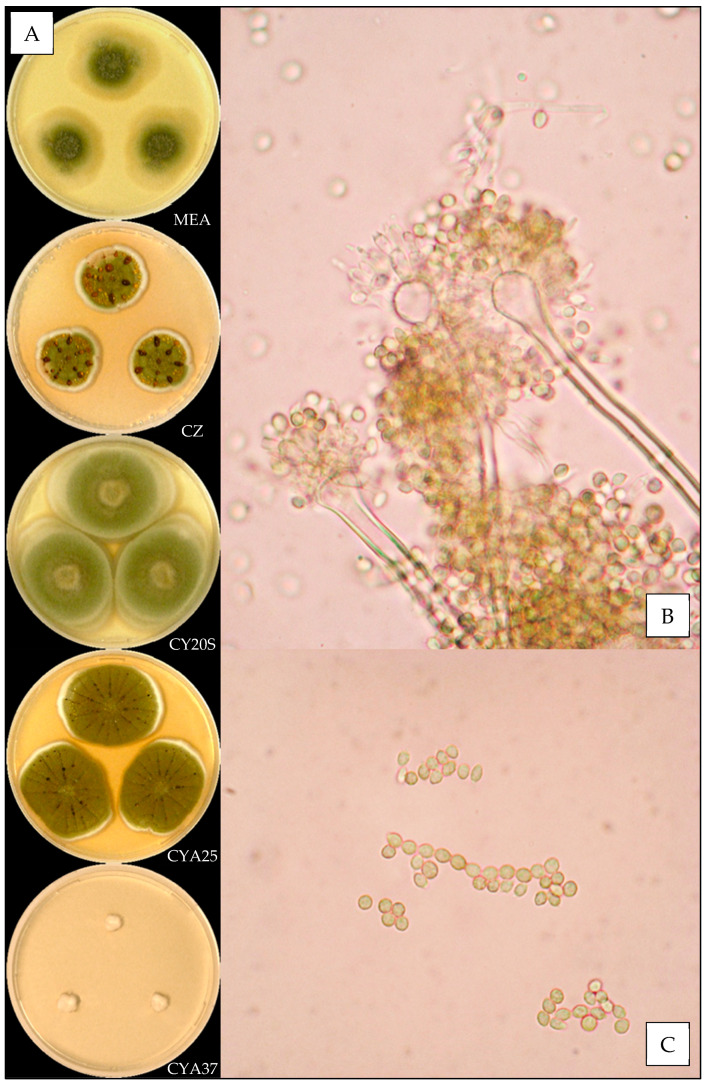
*Aspergillus fructus*: (**A**) from top to bottom, colonies on MEA, CZ, CY20S, CYA25 and CYA37, 14 days. (**B**) Conidial head and (**C**) conidia (1000×).

Description based on *n* = 1 isolate (Figure 4).

**Macroscopic characteristics**

Colony diameters at 7 days, in mm, were: MEA 16–19, CZ 11–16, CY20S 25–30, CYA25 21–27 and CYA37 0–5.

On MEA, there were circular and flat colonies with an entire margin, conidia greyish green (#5a6336), mycelium was white to buff, reverse uncolored, texture was granular and there was no exudate or soluble pigment. On CZ, there were circular and flat colonies with an entire margin, conidia were greenish (#ac9f37) with inconstant shades of dull pink (#d1aa98), mycelium was white to buff, reverse brownish red (#5c2e04), texture was velutinous to granular, exudate was greenish yellow or brownish orange to brownish red (#bdaa30 or #a77d00 to #6a2e00) and there was no soluble pigment. On CY20S, circular and umbonate colonies sometimes radially sulcate with an entire margin, conidia were greenish (#a89950), mycelium was white or buff, reverse dull orange (#d3b072), texture was velutinous to granular and there was no exudate or soluble pigment. On CYA25, circular and flat colonies often radially sulcate with an entire margin, conidia were greyish green (#78681d), mycelium was white to buff, reverse brownish orange (#a35f00), texture was velutinous to granular; when present, exudate was brown (#553400), and there was no soluble pigment. On CYA37, when present, colonies were white and wrinkled and flat with an entire margin, reverse brownish yellow (#b59e73), texture was velutinous and there was no exudate or soluble pigment.

**Microscopic characteristics**

Conidial heads radiate uncolored to greyish green vesicles expanding into pyriform or spathulate vesicles of 12–13 µm in diameter, biseriate, metulae of 4–6 µm covering two thirds to all of the vesicles and phialides of 5–6 µm. No diminutive conidial heads were observed for *Aspergillus fructus* isolates. Conidia of 3–4 µm in diameter were mostly globose or globose to sub-globose, with smooth or finely roughened walls. No Hülle cells or chlamydospores were observed.

#### 3.3.4. *Aspergillus jensenii*

**Figure 5:**
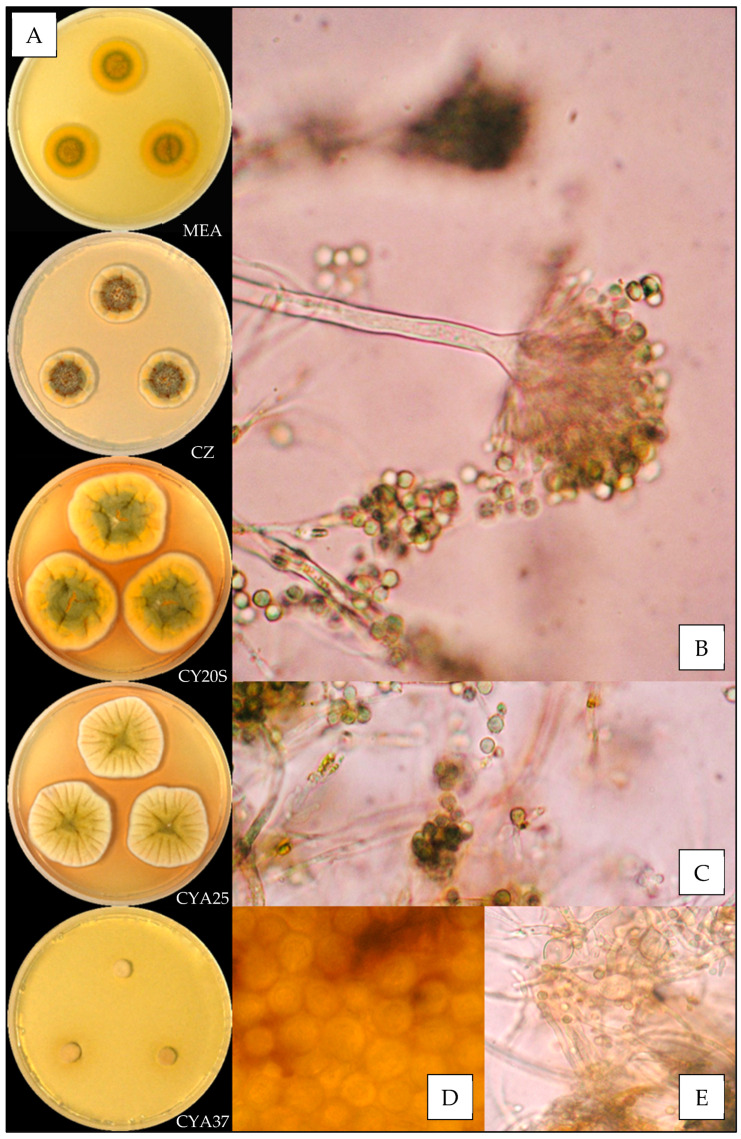
*Aspergillus jensenii:* (**A**) from top to bottom, colonies on MEA, CZ, CY20S, CYA25 and CYA37, 14 days. (**B**) Conidial head, (**C**) conidia, (**D**) globose Hülle cells and (**E**) chlamydospores (1000×).

Description based on *n* = 37 isolates (Figure 5).

**Macroscopic characteristics**

Colony diameters at 7 days, in mm, were: MEA (0–) 3–13 (-18), CZ (6–) 8–14 (–16), CY20S (11–) 16–25, CYA25 (8–) 12–18 (–20) and CYA37 0–5 (–8).

On MEA, there were circular and flat colonies with an entire margin, white to yellow (#eec64e), mycelium was white, reverse uncolored to orange (#f6b54f), texture was granular and there was no exudate or soluble pigment. On CZ, there were circular and flat colonies with an entire margin, conidia were greenish (#a5aa79), mycelium was white, reverse brownish pink to brownish red (#d39f72 to #68260f), texture was granular; when present, exudate was orange to brownish orange (#fa6a00 to #b9611a), and when present, soluble pigment was beige to brownish orange (#e1b589 to #c78107). On CY20S, circular and flat colonies radially sulcate with an entire margin, conidia were pale yellow to dull green (#f2dd9e to #a2a462), mycelium was white, reverse orange red (#9c3d17), texture was velutinous and there was no exudate; when present, soluble pigment was orange red to brown (#b54400 to #341000). On CYA25, there were circular and flat colonies with an entire margin, conidia were greyish green (#a7ac75) with non-constant pale orange shades (#d9bd81), mycelium was white, reverse dark orange to brown (#bf6327 to #6f4215) and texture was velutinous; when present, exudate was orange pink to brown (#fbbe82 to #644533), and when present, soluble pigment was orange (#c66927). On CYA37, when present, there were circular and convex white colonies with an entire margin, reverse brownish yellow (#e0ad5a), texture was velutinous and there was no exudate or soluble pigment.

**Microscopic characteristics**

Conidial heads radiate uncolored to yellow orange vesicles expanding into pyriform or spathulate vesicles of (3–) 7–14 µm in diameter, biseriate, metulae of 4–6 (–7) µm covering two thirds to all of the vesicles and phialides of 4–7 (–8) µm. Diminutive conidial heads were observed for *Aspergillus jensenii* isolates. Conidia of (2–) 3–4 (–5) µm in diameter were mostly globose or globose to sub-globose, with mostly roughened or finely roughened walls. Hülle cells (12–16 µm) and chlamydospores were observed.

#### 3.3.5. *Aspergillus protuberus*

**Figure 6:**
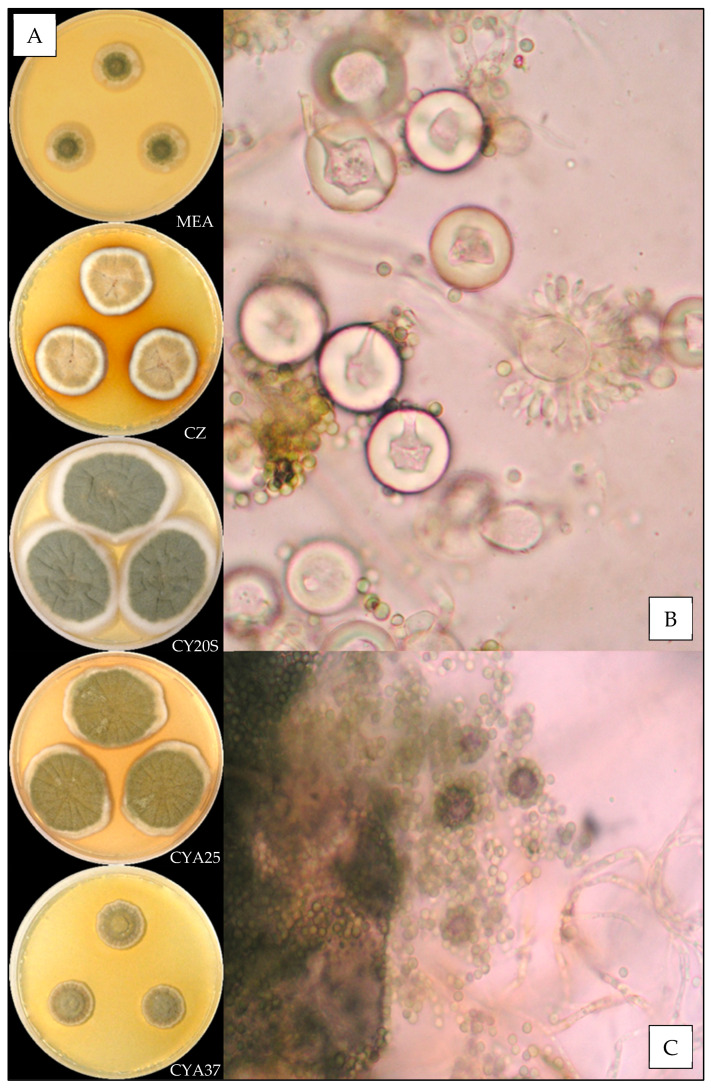
*Aspergillus protuberus*: (**A**) from top to bottom, colonies on MEA, CZ, CY20S, CYA25 and CYA37, 14 days. (**B**) Conidial head and Hülle cells and (**C**) conidia (×1000).

Description based on *n* = 6 isolates (Figure 6).

**Macroscopic characteristics**

Colony diameters at 7 days, in mm, were: MEA 8–18, CZ 5–19, CY20S 22–31, CYA25 16–28 and CYA37 0–5 (–14).

On MEA, there were circular and flat colonies with an entire margin, conidia yellow to greyish green (#e3bc63 to #a0904d), mycelium was white to buff, reverse uncolored to orange (#d5a553), texture was granular and there was no exudate or soluble pigment. On CZ, there were circular and flat colonies with an entire margin, conidia were greyish green or buff (#ac9c56 or #b08d61), mycelium was white, reverse brownish orange (#6b350f), and texture was velutinous; when present, exudate was brown with orange or red shades (#7d4e16 to #531b00), and when present, soluble pigment was pink (#e09a7a) or brownish orange (#a56719). On CY20S, circular and flat or crateriform colonies radially sulcate with an entire margin, conidia were yellowish to greyish green (#ddce61 to #988c62), mycelium was white, reverse brownish orange (#b8671c), texture was velutinous and there was no exudate; when present, soluble pigment was orange to brownish red (#621400). On CYA25, circular and flat colonies often radially sulcate with an entire or undulate margin, conidia were brown to greyish green (from #ae9166 to #aaa581), mycelium was white to buff, reverse brownish orange to brownish red (from #8d531a to #4d2108), and texture was velutinous to granular; when present, exudate was brown (#3e1800), and when present, soluble pigment was orange pink to brownish orange (from #f5b67e to #ac4b00). On CYA37, when present, colonies were dense and convex white to beige (#d3b37c) with an entire margin, reverse brownish yellow (#c5a43b), texture was velutinous and there was no exudate or soluble pigment.

**Microscopic characteristics**

Conidial heads radiate uncolored to greyish green vesicles expanding into pyriform or spathulate vesicles of 11–16 µm in diameter, biseriate, metulae of 3–5 µm covering all of the vesicles and phialides of 4–7 µm. No diminutive conidial heads were observed for *Aspergillus protuberus* isolates. Conidia of 2.5–3.5 µm in diameter were mostly globose to sub-globose, with smooth or finely roughened walls. Hülle cells (14 µm) were found but no chlamydospores were observed.

#### 3.3.6. *Aspergillus puulaauensis*

**Figure 7:**
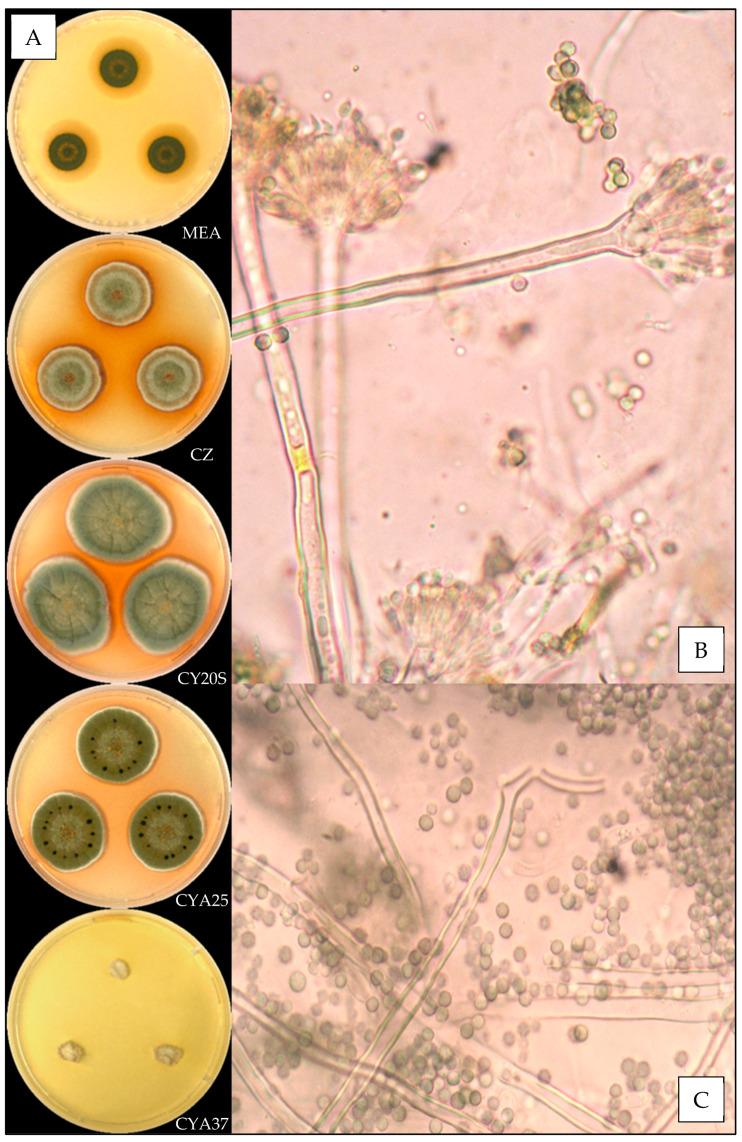
*Aspergillus puulaauensis*: (**A**) from top to bottom, colonies on MEA, CZ, CY20S, CYA25 and CYA37, 14 days. (**B**) Conidial head and (**C**) conidia (1000×).

Description based on *n* = 4 isolates (Figure 7).

**Macroscopic characteristics**

Colony diameters at 7 days, in mm, were: MEA 11–14, CZ 11–15, CY20S 21–25, CYA25 17–19 and CYA37 0–5.

On MEA, there were circular and flat colonies with an entire margin, conidia were dull green to green (#99ab87 to #3f5f36), mycelium was white, reverse uncolored to orange (#a85907), texture was granular and there was no exudate or soluble pigment. On CZ, there were circular and flat colonies with an entire margin, conidia were greyish green (#b6a35b) with orange shades (#daa74e), mycelium was white, reverse brown (#5f412c), texture was granular, exudate was brownish orange (#cc5c00) and soluble pigment was beige to orange (#d78e69 to #d17b19). On CY20S, circular and flat or colonies radially sulcate with an entire margin, conidia were greyish green (#9e8d52), mycelium was white, reverse brownish orange (#8d4d1f), texture was velutinous and there was no exudate; when present, soluble pigment was orange (#db7314). On CYA25, circular and flat colonies radially sulcate with an entire margin, conidia were greyish green to dark green (from #72815c to #354532), mycelium was white, reverse brownish orange (#7f4b1a), and texture was velutinous; when present, exudate was brownish orange to black (#a64601 to #0f0b00), and when present, soluble pigment was orange pink to pale orange (#e3935d). On CYA37, when present, there were dense and convex white colonies with an entire margin, reverse uncolored, texture was velutinous and there was no exudate or soluble pigment.

**Microscopic characteristics**

Conidial heads radiate uncolored to greyish green vesicles expanding into pyriform vesicles of 9–15 µm in diameter, biseriate, metulae of 4–6 µm covering all of the vesicles and phialides of 5–6 µm. No diminutive conidial heads were observed for *Aspergillus puulaauensis* isolates. Globose green conidia were of 3–4.5 µm in diameter, with roughened walls. No Hülle cells or chlamydospores were observed.

#### 3.3.7. *Aspergillus sydowii*

**Figure 8:**
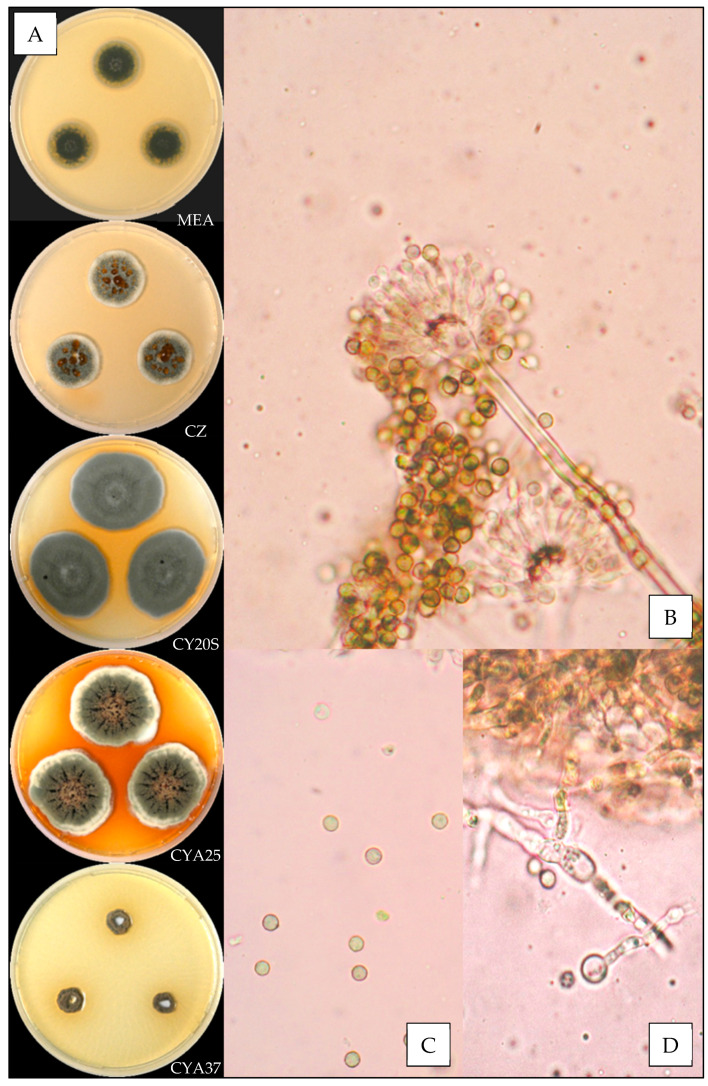
*Aspergillus sydowii*: (**A**) from top to bottom, colonies on MEA, CZ, CY20S, CYA25 and CYA37, 14 days. (**B**) Conidial head, (**C**) conidia and (**D**) chlamydospores (1000×).

Description based on *n* = 2 isolates (Figure 8).

**Macroscopic characteristics**

Colony diameters at 7 days, in mm, were: MEA (11–) 14–21, CZ 12–22, CY20S (14–) 21–34, CYA25 16–26 (–31) and CYA37 0–18.

On MEA, there were circular and flat colonies with an entire margin, conidia were dark blue (#353a40), mycelium was white, reverse uncolored, texture was velutinous to granular and there was no exudate; when present, soluble pigment was orange (#c5792a). On CZ, there were circular and flat colonies with an entire margin, conidia were blue green (#404a45), mycelium was white, reverse brown (#463b2a), and texture was granular; when present, exudate was yellowish to brownish red (#cdac3f to #732f00), and when present, soluble pigment was dull yellow to dull orange (#e5c069 to #c08c35). On CY20S, circular and flat or colonies sometimes radially sulcate with an entire margin, conidia were greyish blue (#5f6a65), mycelium was white, reverse brownish orange (#975f04), and texture velutinous; when present, exudate was black (#12110c), and when present, soluble pigment was pale orange (#d9ad4e). On CYA25, there were circular and flat colonies with an entire margin, conidia were greyish blue (#81827a), mycelium was white, reverse brown (#7e5631), and texture was velutinous; when present, exudate was brownish orange to brownish red (#c0761b to #762f00), and when present, soluble pigment was brownish yellow (#c18919). On CYA37, colonies were dense and convex or wrinkled and crateriform with an entire margin, conidia were grey to dark grey (#aca38a to #554e33), sometimes with shades of brown (#8d6d4a), reverse brownish black (#1b1612), and texture velutinous; when present, exudate was brownish orange (#93520e), and there was no soluble pigment.

**Microscopic characteristics**

Conidial heads radiate brown to greyish blue vesicles expanding into globose to spathulate vesicles of 6–12 (–14) µm in diameter, biseriate, metulae of 4–7 µm covering two thirds to all of the vesicles and phialides of 5–7 (–8) µm. No diminutive conidial heads were observed for *Aspergillus sydowii* isolates. Globose conidia were of 3–4 (–5) µm in diameter, with roughened to distinctly roughened walls. Chlamydospores were found but no Hülle cells were observed.

#### 3.3.8. *Aspergillus tabacinus*

**Figure 9:**
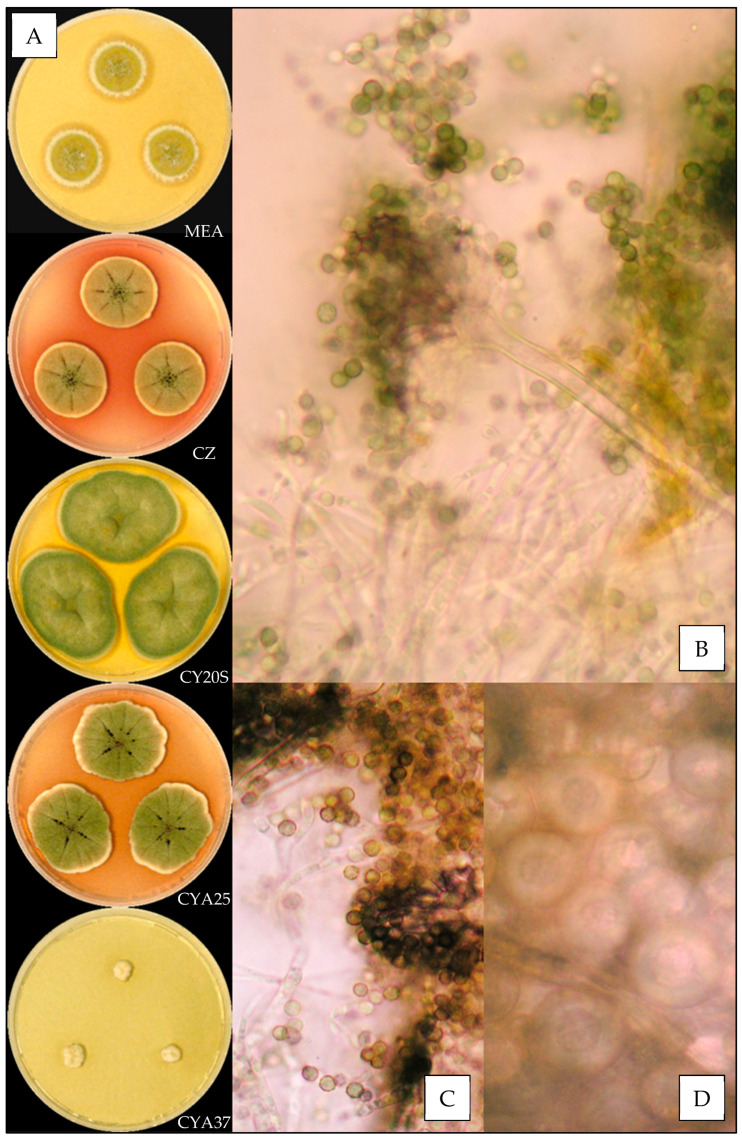
*Aspergillus tabacinus*: (**A**) from top to bottom, colonies on MEA, CZ, CY20S, CYA25 and CYA37, 14 days. (**B**) Conidial head, (**C**) conidia and (**D**) globose Hülle cells (1000×).

Description based on *n* = 2 isolates (Figure 9).

**Macroscopic characteristics**

Colony diameters at 7 days, in mm, were: MEA 13–16, CZ 15–20, CY20S 24–33, CYA25 23–25 and CYA37 0–3.

On MEA, there were circular and flat colonies with an entire margin, conidia were pale greyish green to dark greyish green (from #b1a564 to #565326), mycelium was white, reverse orange (#ce7c11), texture was granular and there was no exudate; when present, soluble pigment was uncolored. On CZ, there were circular and flat colonies with an entire margin, conidia were dark beige to greyish green (#b89656 to #8a7429), mycelium was white to buff, reverse brownish orange (#975409), and texture was velutinous to granular; when present, exudate was yellowish to dark brown (from #d8c96e to #221600), and when present, soluble pigment was pale red orange (#b25128). On CY20S, circular and flat colonies often radially sulcate with an entire margin, conidia were greyish green (#ab934d) with non-constant shades of orange (#dea419), mycelium was white, reverse orange (#e36a15), texture was velutinous and there was no exudate or soluble pigment. On CYA25, circular and flat colonies radially sulcate with an entire margin, conidia were dark beige to greyish green (#e9bc73 to #7d6b14), mycelium was white, reverse brownish orange (#8f3d00), and texture was velutinous; when present, exudate was yellowish to dark brown (from #cfac52 to #0f0b02), and when present, soluble pigment was dull orange (#b2621d). On CYA37, when present, colonies were circular and flat with an entire undulate margin, white, reverse brown (#583306), texture was velutinous and there was no exudate or soluble pigment.

**Microscopic characteristics**

Conidial heads radiate uncolored to greyish green vesicles expanding into pyriform to spathulate vesicles of 9–14 µm in diameter, biseriate, metulae of 4–6 µm covering all of the vesicles and phialides of 5–7 µm. No diminutive conidial heads were observed for *Aspergillus tabacinus* isolates. Globose conidia were of 2.5–3.5 µm in diameter, with distinctly roughened walls. Hülle cells were found but no chlamydospores were observed.

## 4. Discussion

Ninety-three fungal isolates isolated from bioaerosols collected in a cancer treatment center, in contaminated homes and in agricultural environments were identified macroscopically and microscopically as belonging to *Aspergillus* series *Versicolores*. This study constitutes the first report on the distribution of these species in French bioaerosols. Molecular identification by *benA* gene amplification revealed 8 different species among the isolates found in our bioaerosols, which is one of the highest species richness reported to date. Indeed, other studies conducted on isolates collected from indoor environments (air and dust samples) identified by *CaM* gene amplification show that the species richness within the *Versicolores* series ranges from 3 (*Aspergillus creber*, *A. jensenii* and *A. protuberus*) to 11 species (*A. creber*, *A. jensenii*, *A. puulaauensis*, *A. tennesseensis*, *A. venenatus*, *A. amoenus*, *A. fructus*, *A. griseoaurantiacus*, *A. pepii*, *A. protuberus* and *A. sydowii*) [29,39]. *Aspergillus creber* and *A. jensenii* were the most frequently isolated species from French bioaerosols (83% of isolates), which is consistent with studies on *Aspergillus* species belonging to the *Versicolores* series that always find these species, and especially with Jakšić et al.’s study, where these two species represent 62% of the isolates [29,40]. However, we did not find *Aspergillus cvjetkovicii*, *A. griseoaurantiacus*, *A. pepii*, *A. tennesseensis* or *A. venenatus*, which have been isolated from indoor air bioaerosols of the USA and Croatia [26,29,40] but are more frequently found in various foods and feeds and in soil [26,30]. *A. protuberus* and *A. puulaauensis* are not as frequent but remain quite recurrent in indoor air and dust, as indicated by Micheluz et al. and Jakšić et al. [29,39]. *Aspergillus protuberus* was only found in bioaerosols collected in the cancer treatment center with significantly lower relative humidity in comparison to that observed for isolates of other species (*p* = 3.899 × 10^-4^). Nevertheless, no other study on the *Versicolores* series provides information on the climatic conditions during sampling, which does not allow to support if low relative humidity can be considered as a factor facilitating the growth of *A. protuberus*. Measurement of climatic conditions (temperature and relative humidity) during sampling should be systematically performed to provide additional data to explore the fungal ecology of this species. Moreover, all this information could be useful to determine if *Aspergillus protuberus* is an indicator of healthy homes, as opposed to *A. creber*, which is prevalent in damp indoor environments. *Aspergillus sydowii* was only isolated from two bioaerosols collected in contaminated homes, which confirms its presence at a rather low frequency in air compared to food matrices or in human pathology [26,30,31]. *Aspergillus amoenus* was isolated by Jurjevic et al. [26] from different sources (mammary gland, brined meat, *Berberis* sp. fruit) but also from indoor dust in Japan [30], which can explain why we found so few of them among all the collected isolates. This is the first time that *Aspergillus tabacinus* has been isolated from bioaerosols, whereas it is more frequently isolated from tobacco, brined meat or plants (maize) [26]. We also found one isolate of *Aspergillus fructus* which had never been identified in bioaerosols, even though it has already been recovered from dust of water-damaged and control homes in Croatia. However, this species seems more commonly identified from fruits [26]. *Aspergillus versicolor stricto sensu* was not found in our bioaerosols, which confirms the observations of Jurjevic et al. [26] and Kobayashi et al. [30], who only reported its presence in food products (dairy feed, rice and noodles).

We also reported the first observation of globose Hülle cells and the presence of diminutive conidial heads for *Aspergillus creber*, which were not described by Jurjevic et al. [26].

We observed, as previously described [26,38], an important intraspecific macroscopic polymorphism, which requires the use of sequencing to allow an identification at the molecular level for species of the *Versicolores* series. Nevertheless, the most recurrent species *Aspergillus creber* and *A. jensenii* have distinct macroscopic and microscopic aspects: *A. creber* has a grey-green appearance, while colonies, hyphae, conidial heads and conidia of *A. jensenii* are yellow-colored.

Further studies will be conducted to explore the metabolic and toxicological profiles of these different species. An analytical approach using HPLC-MS/MS will allow the identification and quantification of target metabolites, while cytotoxicity assays on lung cells will provide supplementary information on the toxicity of the isolates. All these data will allow a better knowledge of the species belonging to the series *Versicolores* and a better assessment of the exposure to bioaerosols containing these molds and their effects on health.

## Figures and Tables

**Table 1:** Temperature and relative humidity measured during bioaerosols’ collection.

Species	N	Temperature (°C)			Relative Humidity (%)		
		Min	Max	Mean	Min	Max	Mean
*A. amoenus*	1	-	-	19.9	-	-	66.1
*A. creber*	40	9.4	26.1	20.7	23.8	74.3	57.5
*A. fructus*	1	-	-	18.9	-	-	41.2
*A. jensenii*	37	10.0	24.7	20.8	23.8	70.8	50.2
*A. protuberus*	6	22.9	24.3	23.7	26.1	45.7	34.6
*A. puulaauensis*	4	10.3	23.9	20.2	23.8	66.9	53.0
*A. sydowii*	2	22.8	26.1	24.5	46.7	69.6	58.2
*A. tabacinus*	2	18.4	20.8	19.6	32.4	49.2	40.8

**Table 2:** Summary of microscopic characteristics.

	*Aspergilllus* *amoenus*	*Aspergillus* *creber*	*Aspergillus* *fructus*	*Aspergillus* *jensenii*	*Aspergillus* *protuberus*	*Aspergillus* *puulaauensis*	*Aspergillus* *sydowii*	*Aspergillus* *tabacinus*
Conidial head (µm)	12–15	(3–) 5–13 (–15)	12–13	(3–) 7–14	11–16	9–15	6–12 (–14)	9–14
Metulae (µm)	4–6	(3–) 4–6 (–7)	4–6	4–6 (–7)	3–5	4–6	4–7	4–6
Phialides (µm)	5–6	4–6 (–8)	5–6	4–7 (–8)	4–7	5–6	5–7 (–8)	5–7
Conidia size (µm)	3–4	2.5–4.5	3–4	(2–) 3–4 (–5)	2.5–3.5	3–4.5	3–4 (–5)	2.5–3.5
Conidial wall ornementation	finely or distinctly roughened	finely or distinctly roughened	smooth or finely roughened	roughened or finely roughened	smooth or finely roughened	roughened	roughened to distinctly roughened	distinctly roughened
Hülle cells	−	+	−	+	+	−	−	+
Chlamydospores	−	−	−	+	−	−	+	−
Diminutive conidial head	−	+	−	+	−	−	−	−

− absence; + presence.
